# Does proximity to conflict affect tourism: Evidence from NATO bombing

**DOI:** 10.1371/journal.pone.0258195

**Published:** 2021-10-20

**Authors:** Marina Tkalec, Ivan Žilić

**Affiliations:** The Institute of Economics, Zagreb, Zagreb, Croatia; Xiamen University, CHINA

## Abstract

Although conflict, war, violence, and terrorism affect tourism, research that identifies possible channels of these effects is scarce. We explore if the adverse effects are channelled through proximity to conflict areas. We use the conflict in Kosovo in 1999 and the country Croatia as a quasi-natural experiment and take advantage of the specific north-west to south-east orientation of Croatian Adriatic counties to identify the effect of NATO bombing in Kosovo on tourism outcomes as well as the potential proximity channel. Using data on the population of Croatian firms and the difference-in-differences identification strategy we find that tourism companies’ revenues decreased significantly due to NATO bombing, especially in accommodation services and in companies with 50 or more employees. However, using a synthetic control approach we find that the adverse effect is only transitory. Analysing heterogeneous effects with respect to the distance of the firm from Kosovo—using a linear and a more flexible model—we find compelling evidence that within-country proximity to conflict is not a significant channel through which the negative effect propagates.

## 1 Introduction

Tourism—an activity largely based on pleasant experiences—is fundamentally incompatible with conflict, war, violence, and terrorism. While the 21st century has so far been statistically the most peaceful one, numerous violent conflicts and comprehensive media coverage have brought dreadful events into the cities and homes of many people. For example, according to the [1] the number of countries experiencing a terrorist attack in 2015 amounted up to 92 with 29,376 people dying during these terrorist events. When wars and other sorts of conflict are added, the number goes up by a high degree. While these horrific events are important issues on their own, it remains to see how they affect tourism. Recent crisis events in popular tourist destinations—for example, attacks in Tunisia, Turkey, France and Spain—might shift tourist demand towards places which are perceived safer.

While the literature on the nexus between violent conflict and tourism is (unfortunately) growing, the empirical evidence on the channels of these adverse effects is scarce. In this paper, using a dataset on the universe of Croatian firms and the difference-in-differences identification strategy, we explore how the NATO bombing of Kosovo and the rest of the Yugoslavian (note that in 1999, Yugoslavia was composed of Serbia and Montenegro only, and that Kosovo was a part of Serbia [2–4]) territory in 1999 affected revenues of firms in the tourism sector in Croatia—which, at the time, was a neighbouring country of the conflict-infected Yugoslavia.

Given that firm-level data contain information on firm location, we are able to explore not only the magnitude, sign, significance and persistence of the effect, but also analyse a potential channel of within-country proximity to conflict. In particular, we are interested if the adverse effect is stronger if the firm in the tourism sector is closer to the bombing site. In order to answer this question we use the north-west to south-east orientation of the seven Croatian Adriatic counties. The utmost north county, Istria, is less than 100 km away from Venice, while the utmost south Dubrovnik-Neretva county bordered with the Federal Republic of Yugoslavia and was only 160 km from Kosovo (where most of the NATO bombings took place). If a heterogeneous effect with respect to proximity is established—tourism firms closer to conflict (ones on the south) suffered more in 1999—we can argue that within-country proximity to conflict is a significant channel of this adverse effect.

We find compelling evidence that revenues of firms in the tourism sector in Croatia decreased significantly due to NATO bombing, especially in accommodation services and in companies with 50 or more employees. The magnitude of the effect is around -16.6 percent and this result is stable across numerous robustness checks. In addition, applying the synthetic control approach from [5], we find the the adverse effects was only temporary. However, constructing a measure of distance between the municipality of the firm’s headquarters and NATO bombing site (Kosovo), and using this proximity as an intensity of exposure to conflict, we do not find any evidence that closer firms experienced more adverse effects. Results of both linear and a more flexible specification yield the same conclusion: within-country proximity to conflict does not significantly affect the magnitude of the negative effect.

The contribution is twofold. First, we document the effect of the Kosovo conflict and the NATO bombing in 1999 on Croatian tourism thus following the line of literature established in [6–11]. Second, we use the conflict in Kosovo and the country of Croatia as a quasi-natural experiment and take advantage of the specific north-west to south-east orientation of Croatian Adriatic counties to identify and estimate a potential within-country proximity channel. While the results established in this paper are certainly context—i.e. Croatian-specific, they argue that, given the violent conflicts in popular tourist destinations, this research can facilitate understanding of negative effects of conflict on tourism and their channels.

The rest of the paper is organised as follows. In the second section we review the literature, from conflict and terrorism studies to tourism research, in order to motivate our research idea, while section three provides details on the NATO bombing of Yugoslavian territory. Section four describes the data, methodology, and the identification strategies, while in section five we present our baseline results. This is followed by a discussion on the channel of adverse effects, while the last section concludes the paper.

## 2 Tourism and conflict

Existing tourism and conflict literature mostly just documents events that cause tourism to tumble and on occasion tries to measure the size of the effect, the geographical reach, and the time it took tourism to stabilize after the unagreeable event(s). However, a large body of research focuses on tourism management in periods of crisis, conflict, and war. For example, [10–12] explore the tourism of Bali, while [13–15] focus on Israel. Other Asian case studies include [16] for Nepal and [17] for the Persian gulf, while [18] analyse tourism development challenges in post-conflict sub-Saharan Africa. Finally, there are three studies done for Europe, [19, 20] for Cyprus and [6] for Bosnia and Herzegovina.

[7] argue that a terrorist attack in Spain caused tourist arrivals to drop by more than 140,000 and that the effects lasted for at least three months [8]. Explore a number of terrorist attacks over a long period of time. They show that terrorist attacks deter tourism as revenues in the tourism industry fall even six to nine months after the attack [21]. Describes the impacts of the September 11 terrorist attacks on the US economy. He argues that the tragedy had been particularly adverse for the airline industry and the manufacture of airplanes. The hotel industry was also seriously damaged as during the first three months hotel bookings declined by 20 to 50 percent. Other economic sectors that were also hit were casinos, sports tourism, etc. He also found that some states suffered more than others as tourism is more important for their economy (e.g. Florida, California, and Nevada) [22]. Analyse terrorism events and their impact on tourist destinations and the tourism industry. They find that in three-quarters of terrorist events that occurred between 1985 and 1998, the acts caused a decline in tourism demand with the median length of 1 to 3 months, with more than one-third causing a decline of 4 to 6 months. On a broader note, Wall (1996) finds that instability and violence affect not only locations under conflict but also wider parts stretching to the whole region, country and neighbouring countries.

The only study in tourism research that uses firm-level data and explores the influence of terrorist events is the one by [9] who build a supply and demand model of the Israeli hotel industry where they measure how foreign tourists respond to terrorist events. Their results, although only marginally reflecting the effects of terrorism on tourism, show there is a large and statistically significant negative effect of terrorism in Israel on foreign demand quantity measured by overnight stays. Our research is probably closer to [23], who were the first to use firm-level data to study effects of war in Sierra Leone. They use the geographical variation in the magnitude of conflict to estimate the effect of violence on firm growth. They find that firms in areas that were more exposed to civil war were smaller firms.

## 3 Background of the NATO bombing of Yugoslavian territory

**Fig 1:**
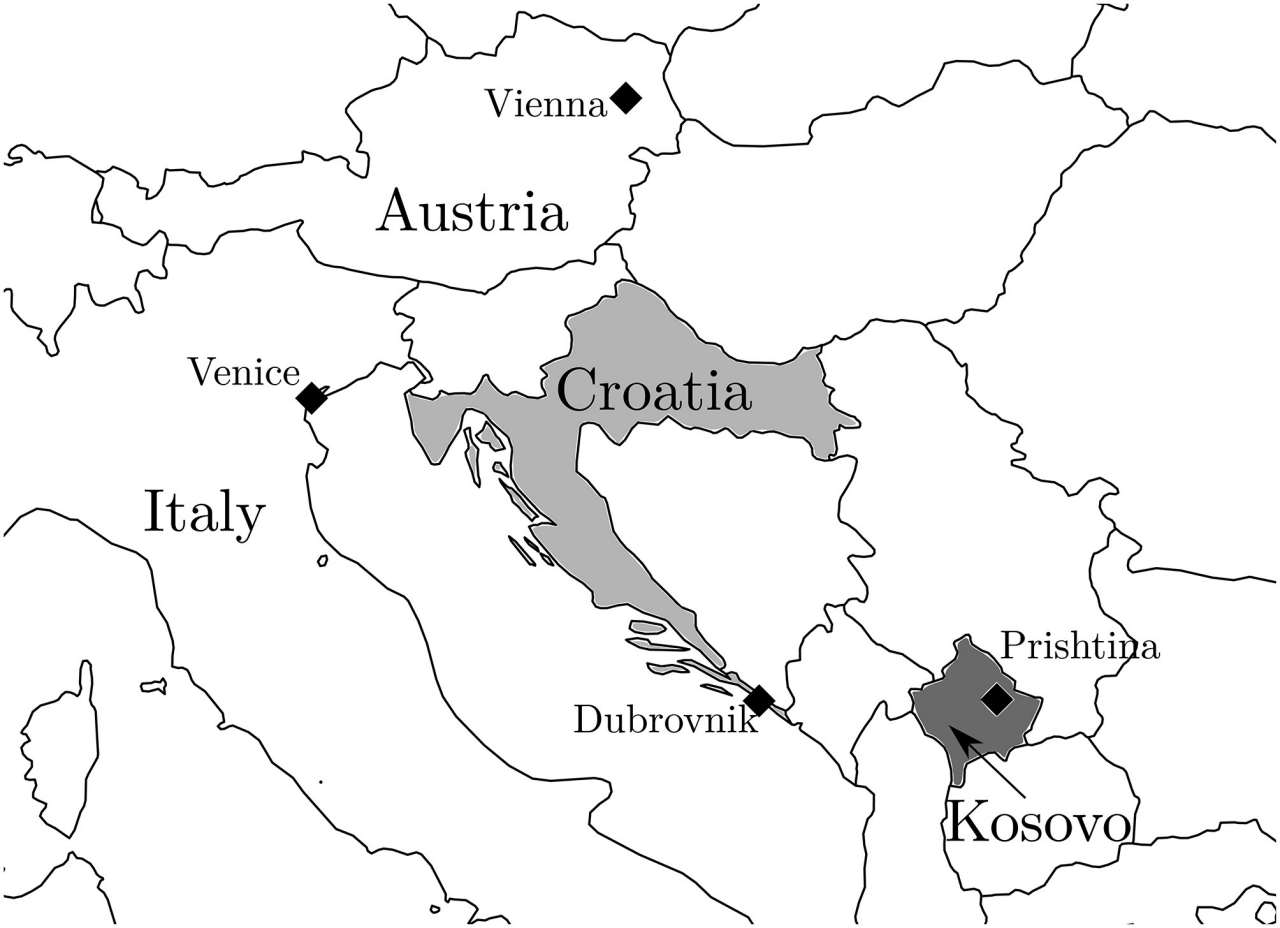
Map of affected and surrounding areas. Source for original data: Google Maps.

In the spring of 1998, the autonomous province of Serbia—Kosovo—populated by up to 90 percent ethnic Albanians, was hit by conflict between the Albanian guerilla movement, the Kosovo Liberation Army, and the Serbian police. In the aftermath of the demise of Yugoslavia and wars in Croatia and Bosnia and Herzegovina, the UN was on standby to stop a new war in the region. After the mediation broke down, in March 1999, NATO started the three-month long aerial bombardment of Yugoslavian territory (mostly Kosovo and Serbia). In June 1999, NATO and the Federal Republic of Yugoslavia agreed a withdrawal of Serb forces from Kosovo [24–29]. At the time, Croatia directly bordered with the Republic of Serbia on the east of the country, while on the south, the Dubrovnik-Neretva county bordered with the Republic of Montenegro (Fig 1).

**Fig 2:**
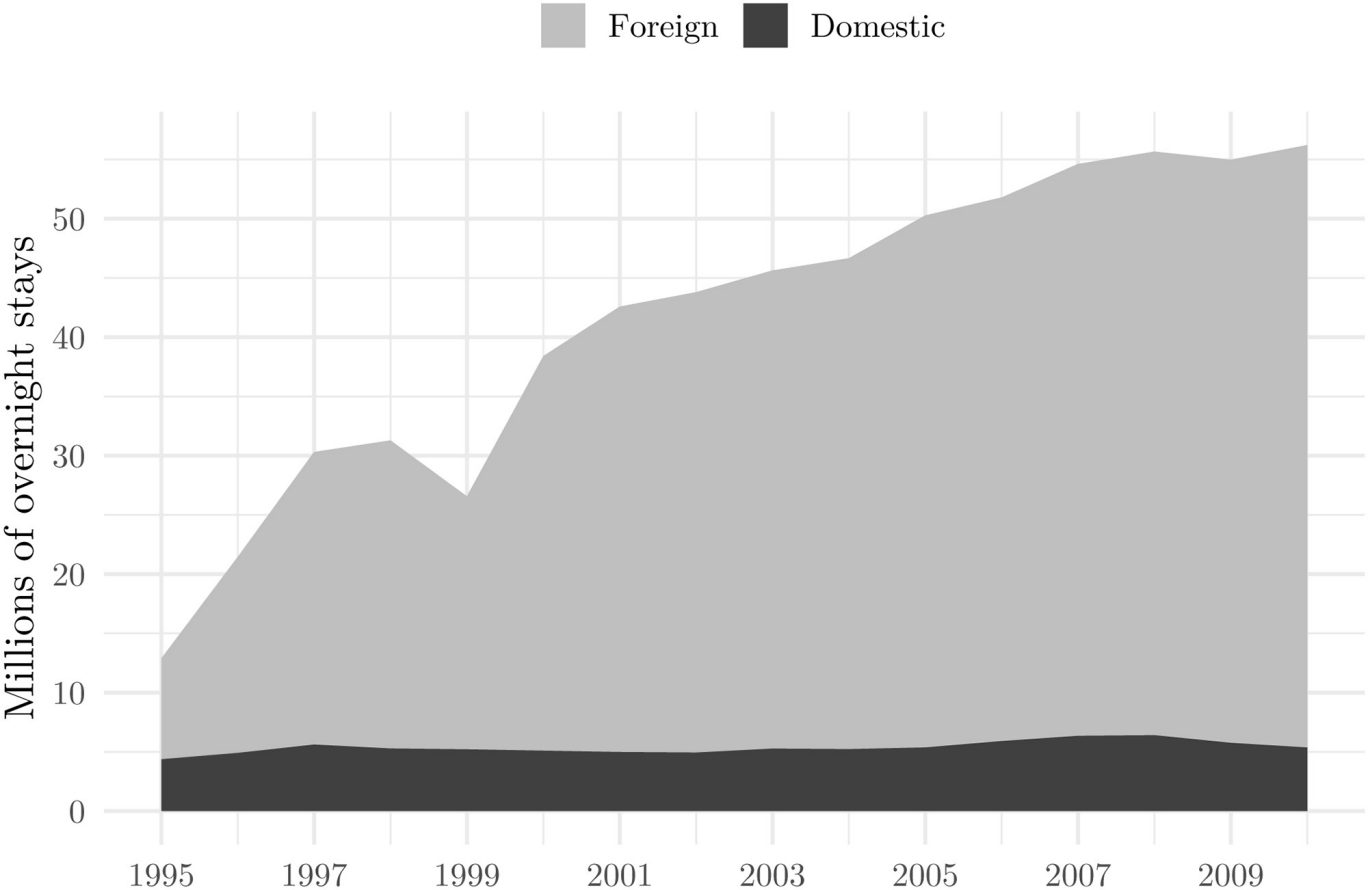
Tourist nights: Foreign and domestic tourists. Source: Croatian Bureau of Statistics.

Although there was no direct border with the autonomous province of Kosovo, the instability in the region at the time influenced the Croatian economy to a large extent, tourism in particular. The conflict captured international attention. For example, The Economist featured a story on the bombings, while the [30, 31] warned against possible adverse effects on Croatians tourism. And indeed, aggregate data suggest that tourist arrivals in 1999 dropped by 725 thousand and overnight stays by 4.726 million (almost entirely attributed to foreign tourists, which can be seen from Fig 2) when compared to the previous year. The following tourist season, 2000, in which the Kosovo-Serbia conflict ceased and 12 months after the NATO bombing, the number of tourist arrivals and overnight stays not only surpassed the level of 1999, but overshot the level of 1998, resulting in an annual increase in overnight stays amounting up to 12.057 million. To sum up, after the end of the war in Croatia in 1995, arrivals and overnight stays have been growing, except in 1999. When exploring possible reasons for such an irregularity in the data, one can think only of the NATO bombing of Serbia as it is a known fact that instability, wars, and terrorism significantly affect tourism. Data for other economic sectors in Croatia at the time do not share the same pattern, which is reason more in favour of our hypothesis.

## 4 Methodology and data

In order to estimate the effect of bombings on tourism, we use a universe of limited liability firms in Croatia across the 1993–2008 period, obtained from annual balance sheets and profit and loss reports, self-reported for the tax authorities. The dataset contains firm ID, NACE industry codes, county of headquarters, year of incorporation and exit, complete balance sheets, and profit and loss statements. The dataset has a longitudinal component as we can follow a particular firm throughout time. Given that we have access to the pre- and post-bombing period, we can identify the magnitude as well as the persistence of effect. First, we establish the effect of NATO bombing on the whole Croatian tourism sector, using two strategies: difference-in-differences [32] and synthetic controls [5].

Using the 1993—1999 subsample and the difference-in-differences strategy we estimate the short-term effect of NATO bombings on Croatian tourism. Intuitively, DD compares the differences between outcomes of a treated unit—in our case revenues of firms in the tourism sector—before and after the treatment (NATO bombing), with the outcomes of control units—firms in the manufacturing sector—before and after the treatment. Using this method we are able to control for the overall momentum of economic activity, thus extracting only the causal effect of NATO bombing on tourism.

More formally, we estimate the following equation:

$$\begin{array}{c} Y_{istl}=\alpha +\beta \;treat_{is}+\gamma \;post_{it}+\delta_{DD}\;\left(treat_{is}\times post_{it}\right)+\phi^{\prime }X_{it}+\zeta_{l}+\eta_{s}+e_{istl} \end{array}$$ (1)

where:

- *Y*_*istl*_ is the *log*(1+*revenues*) of a firm *i* in sector *s* in time *t* in location *l*.
- *treat*_*is*_ is an indicator if a firm *i* is in sector *s* which is treated. If a firm is in the tourism sector, *treat*_*is*_ takes value 1, and if the firm is in the manufacturing sector, *treat*_*is*_ takes value 0; therefore *β* reflects differences in log revenues between the two sectors.
- *post*_*it*_ is an indicator if a firm *i* is operating in treated time *t*. If a firm is operating in 1999, *post*_*it*_ takes value 1, and 0 otherwise; therefore *γ* reflects a common time change in log revenues of both sectors.
- *treat*_*is*_ × *post*_*it*_ is an interaction term which identifies the treatment. If a firm *i* is in the tourism sector in 1999, this interaction takes the value 1, and 0 otherwise—if any of the two conditions is not met. Hence *δ*_*DD*_ is the causal parameter of interest—effect of NATO bombing on log revenues of firms in the tourism sector.
- **X**_*it*_, *ζ*_*l*_ and *η*_*s*_ are time-variant firm-level controls (employment, assets, and debt), location fixed effects, and sector fixed effects, respectively.

**Fig 3:**
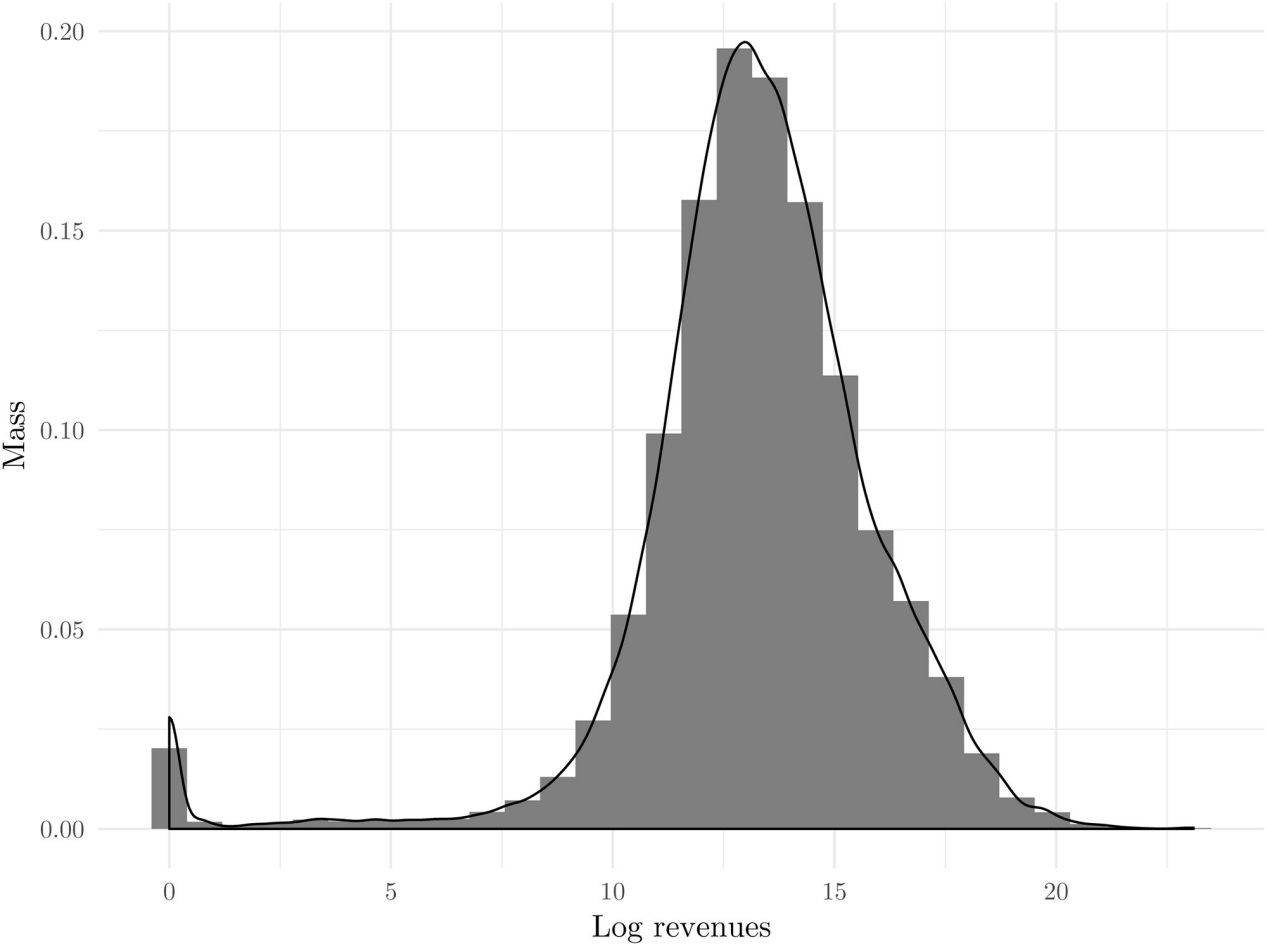
Histogram of log revenues. Source: The Institute of Economics, Zagreb.

Table 1 presents the summary statistics, while Fig 3 presents the histogram of log revenues of data used in this estimation.

**Table 1:** Descriptive statistics.

	*1993–1999 sample (N = 38,578)*		*1998–1999 sample(N = 14,177)*	
	Mean	Std.dev.	Mean	Std.dev.
Log revenues	13.16	2.91	13.16	2.95
Treated sector	0.18	0.38	0.19	0.39
Treated period	0.19	0.39	0.52	0.50
Distance from Kosovo (in km)	514.58	87.75	514.95	88.63
Employees	26.67	90.35	23.55	81.90
Accommodation	0.04	0.21	0.04	0.21
Hotels	0.03	0.18	0.03	0.18
Vacation establishments	0.005	0.07	0.01	0.08
Camping sites	0.004	0.06	0.005	0.07
Other accommodation	0.002	0.04	0.002	0.04
Food and beverage	0.13	0.34	0.15	0.35
Restaurants	0.05	0.21	0.06	0.23
Catering	0.003	0.06	0.004	0.06
Beverage service	0.08	0.28	0.09	0.28
Adriatic region	0.32	0.47	0.33	0.47

In order to explore the long-run consequences of bombing and use other sectors apart from manufacturing as a potential control, we also use a synthetic control approach introduced by [5]. Synthetic control argues that a combination of units constitutes a betters control group for the unit exposed to the treatment than any single unit alone. This combination is a proxy for what the single unit would have experienced in the absence of the treatment. In our case, the treatment group is the tourism sector, while other sectors build the synthetic group. The synthetic tourism sector is constructed as a weighted average of the control sectors represented by the remaining 18 sectors. The weights are determined so that the synthetic tourism sector is most likely to resemble that sector in case there was no NATO bombing at the time. This is achieved using a set of predictors, same as in the DD estimation, of tourism sector outcomes in the years before the treatment. Other examples of the application of the method can be seen in [33–36].

### 4.1 Identification

A crucial assumption for identification of causal effects of NATO bombing on Croatian tourism outcomes is the parallel trend assumption. Intuitively, treated units—firms in the tourism sector—and control units—firms in the manufacturing sector—should have parallel pretreatment trends in terms of outcome. If this assumption holds, one can infer what an outcome of a counterfactual treated unit—outcome of a treated unit had the treatment not occurred—would look like. Therefore, the estimation of a causal effect would be a simple comparison of the outcome of the treated unit and the counterfactual outcome of the treated unit.

**Fig 4:**
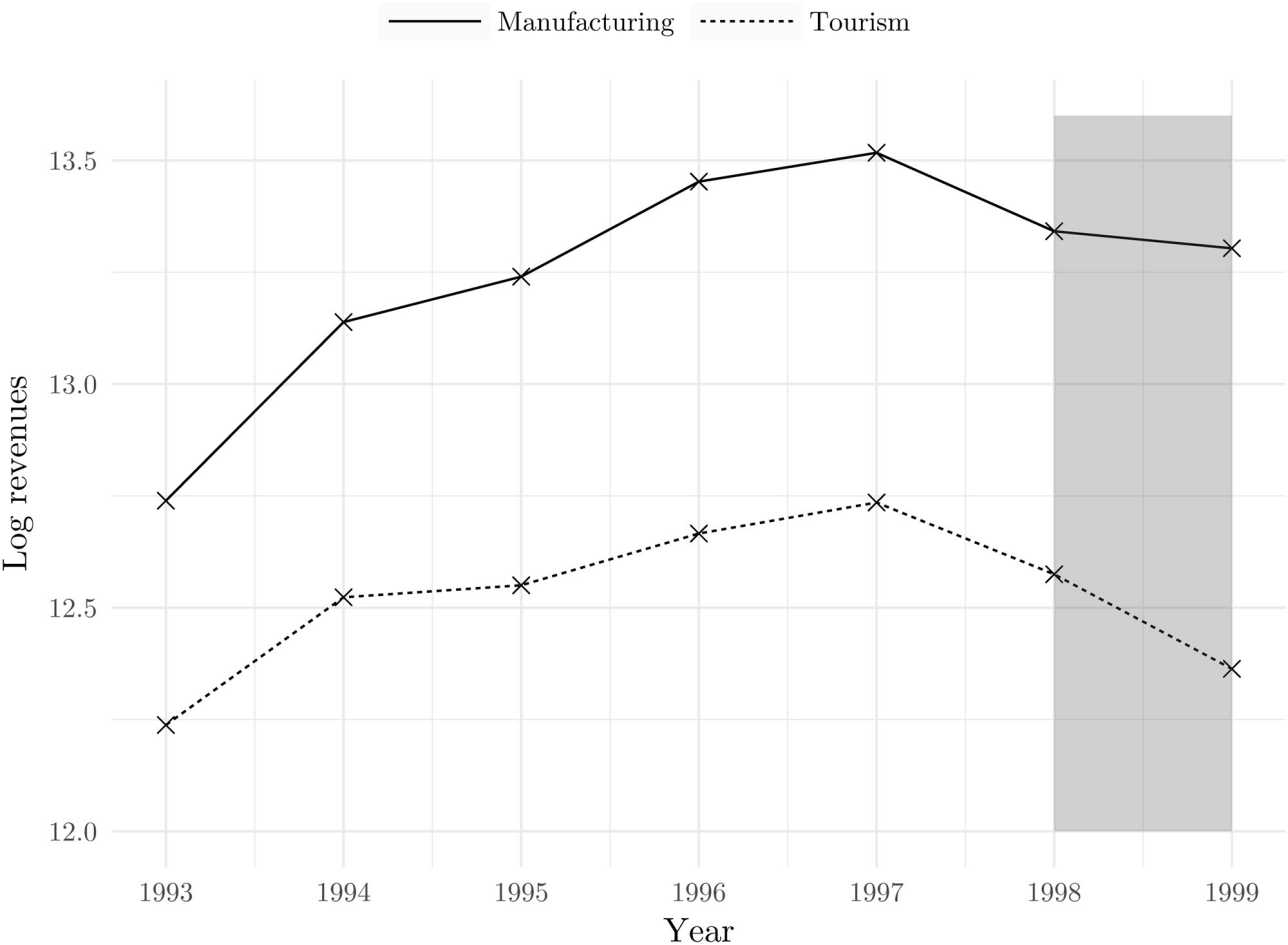
Revenues of manufacturing and tourism industries. Source: The Institute of Economics, Zagreb.

**Fig 5:**
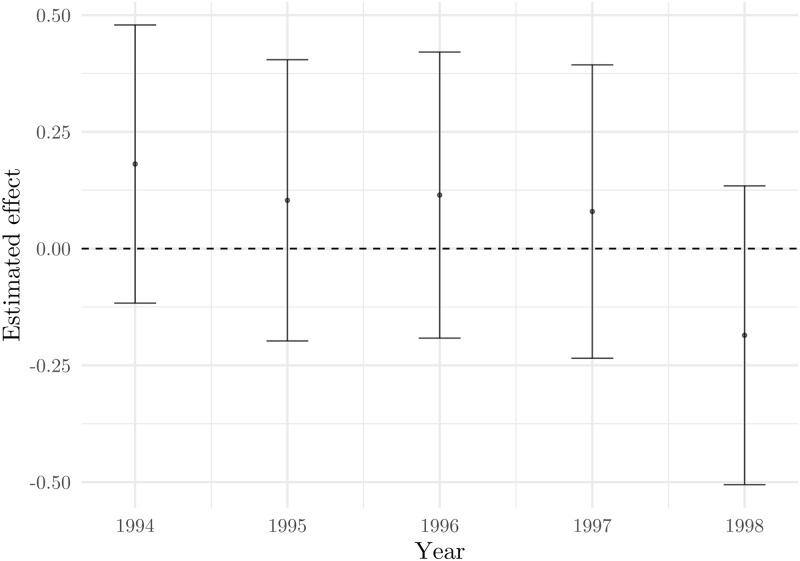
Parallel trends assumption test. Notes: Dots present point estimates of time-specific difference in outcomes between a firm in the treated and in the non-treated sector (tourism vs. manufacturing). The bars present 95% confidence intervals based on standard errors clustered at the firm level.

We take firms in the manufacturing sector as control units as we argue that manufacturing should not be affected directly by the NATO bombing. In order to reinforce the decision to take manufacturing as the nontreated sector, we show Fig 4 which clearly indicates that firms in tourism and manufacturing do follow a pretreatment parallel trend in terms of log revenues. Furthermore, Fig 5 shows more formally that parallel trends assumption is likely to hold: the coefficients next to the interaction terms of prebombing time dummies and the indicator for the treated sector are not statistically significant.

Although the validity of parallel trends enables us to estimate the causal effect of interest, we argue that the estimated object is in fact a lower bound of the effect of NATO bombing on Croatian tourism. Although the manufacturing sector should not be affected directly by war proximity, there are a number of general-equilibrium channels which could adversely affect the manufacturing businesses. For example, lower revenues from tourism might decrease internal demand for manufacturing products. Therefore, if the control units are adversely affected by the NATO bombing, then the estimated effect is actually lower than the true effect. Hence, as we argue that war proximity did not affect manufacturing in a positive way, the true effect of NATO bombing on tourism-firm performance is at least equal to the estimates we present.

Our identification strategy also uses two interesting characteristics of the quasi-experiment we explore. First, the NATO bombing of Serbia due to the Kosovo conflict happened exactly three months before the peak of the tourist season, from March to June 1999. This was a one-off event that appeared only in 1999 and that could affect only the tourist season of 1999. In the years before and after the conflict, tourism was growing steadily. Second, Croatian Adriatic counties (in which most of Croatian tourism is concentrated) have an interesting geography. The seven counties are stacked one on top of another starting with Istria on the north-west, reaching all the way to the Dubrovnik-Neretva county on the south-east. The distance between the utmost north and the utmost south point is more than 450 kilometres, which gives us substantial variation in proximity to NATO bombing sites (Fig 1).

These two characteristics enable an analysis of not only the size of the effect for different firms but also an analysis of potential channels through which conflict operates. Mainly, we are interested if the adverse effects are channelled through geographical proximity to the conflict-inflicted area.

## 5 Results

### 5.1 Difference-in-differences

In this section we establish the effect of NATO bombing on revenues of firms in the tourism sector. Table 2 presents the results of estimating Eq 1 using different model specifications and time frames. In particular, models in columns (1) to (3) use a larger data sample—from 1993 to 1999, while models in columns (4) to (6) use a two-period model with only 1998 and 1999 data. Within the specific time frame, the models differ in terms of covariate inclusion: models in column (1) and (4) use no covariates, (2) and (5) include location and subsector fixed effects, while models in column (3) and (6) also include the number of employees, assets and debt (which could be viewed as bad controls as they could be affected by the treatment—bombing). We present results from different specifications in order to automatically embed robustness checks, but our preferred specification is presented in column (5).

**Table 2:** Effect of Kosovo bombings on revenues in tourism sector.

	*Dependent variable: log revenues*					
	*1993–1999 sample*			*1998–1999 sample*		
	(1)	(2)	(3)	(4)	(5)	(6)
Treated sector x Treated period	−0.234***	−0.191**	−0.203***	−0.173**	−0.166**	−0.180**
	(0.076)	(0.076)	(0.074)	(0.070)	(0.071)	(0.071)
Treated sector	−0.706***	−0.643***	−0.587***	−0.767***	−0.793***	−0.739***
	(0.078)	(0.152)	(0.147)	(0.091)	(0.182)	(0.176)
Treated period	0.008	0.036	0.035	−0.038	−0.029	−0.001
	(0.027)	(0.028)	(0.026)	(0.027)	(0.027)	(0.027)
Employment	—	—	0.011***	—	—	0.012***
			(0.0004)			(0.001)
Long-term assets	—	—	0.000	—	—	0.000*
			(0.000)			(0.000)
Long-term debt	—	—	0.000***	—	—	0.000*
			(0.000)			(0.000)
Municipality fixed effects	No	Yes	Yes	No	Yes	Yes
3-digit NACE code effects	No	Yes	Yes	No	Yes	Yes
Observations	38,463	38,463	35,482	14,177	14,177	14,131

Note: Standard errors clustered at the firm level are in brackets.

*p<0.1;

**p<0.05;

***p<0.01

Table 2 provides compelling evidence of severe significant adverse effect of NATO bombing on revenues of firms in the tourism sector. For example, our preferred specification, presented in column (5), indicates that tourism experienced a 16.6 percent drop in revenues due to NATO bombing. This effect is statistically significant at the 5 percent level and other specifications indicate that the effect is indeed adverse and significant, ranging from -16.6 percent to -23.4 percent. As already mentioned in section 4.1, we view this estimate as the lower bound of the true effect as the control units—firms in the manufacturing sector—could also be adversely affected by NATO bombing via general-equilibrium channels. These inferential conclusions hold even if we cluster standard errors on a different level. For example, clustering standard errors on the three digit NACE sector, on the county, municipality, municipality and three digit NACE sector level yields the same set of conclusions. You can find these results in the Table 7 in S1 Appendix.

In order to document the effect in more detail, we also explore whether this overall effect is coming either from: (i) accommodation or (ii) food and beverage service activities (which are subcategories of the tourism sector according to the Croatian NACE classification). Tables 3 and 4, which present results using our preferred specification (Model (5)), indicate that overall adverse effects came mostly from accommodation activities, especially hotels, where revenues dropped by 33.6 percent. On the other hand, the effect on food and beverage service activities, although negative, is not statistically significant. The negative effect on beverage activities, i.e. bars and coffee shops, is statistically significant though, implying that adverse effects are dominantly coming from hotels, and alternatively from beverage activities. The result that the adverse effect in tourism is driven by a drop in revenues of accommodation business and not restaurant businesses could probably be explained by the fact that accommodation establishments are mostly frequented by foreign tourists while restaurants are visited by both domestic and foreign tourists, and for reasons other than tourism, e.g. business, leisure, everyday nourishment.

**Table 3:** Heterogeneous results: Accommodation and subcategories.

	*Dependent variable: log revenues*				
	All types of accommodation	Hotels	Vacation establishments	Camping sites	Other accommodation
Treated sector x Treated period	−0.293**	−0.336**	−0.236	0.351	−0.572
	(0.137)	(0.159)	(0.385)	(0.316)	(0.731)
Treated sector	0.632***	1.466***	−1.785***	−0.473	−0.353
	(0.227)	(0.230)	(0.601)	(0.694)	(0.608)
Treated period	−0.030	−0.029	−0.028	−0.028	−0.028
	(0.027)	(0.027)	(0.027)	(0.027)	(0.027)
Municipality fixed effects	Yes	Yes	Yes	Yes	Yes
Observations	12,094	11,908	11,553	11,526	11,490

Notes: Estimation includes the 1998–1999 period for all models. Columns two, three, four and five represent subcategories of the first column. Standard errors clustered at the firm level are in brackets.

*p<0.1;

**p<0.05;

***p<0.01

**Table 4:** Heterogeneous results: Food and beverage service activities and subcategories.

	*Dependent variable: log revenues*			
	All food and beverage service activities	Restaurants	Catering and other food service activities	Beverage service activities
Treated sector x Treated period	−0.113	−0.015	0.237	−0.195**
	(0.079)	(0.140)	(0.273)	(0.094)
Treated sector	−1.032***	−0.804***	−0.610	−1.174***
	(0.103)	(0.170)	(0.686)	(0.121)
Treated period	−0.028	−0.028	−0.027	−0.028
	(0.027)	(0.027)	(0.027)	(0.027)
Municipality fixed effects	Yes	Yes	Yes	Yes
Observations	13,544	12,241	11,520	12,705

Notes: Estimation includes the 1998–1999 period for all models. Columns two, three, four and five represent subcategories of the first column. Standard errors clustered at the firm level are in brackets.

*p<0.1;

**p<0.05;

***p<0.01

**Fig 6:**
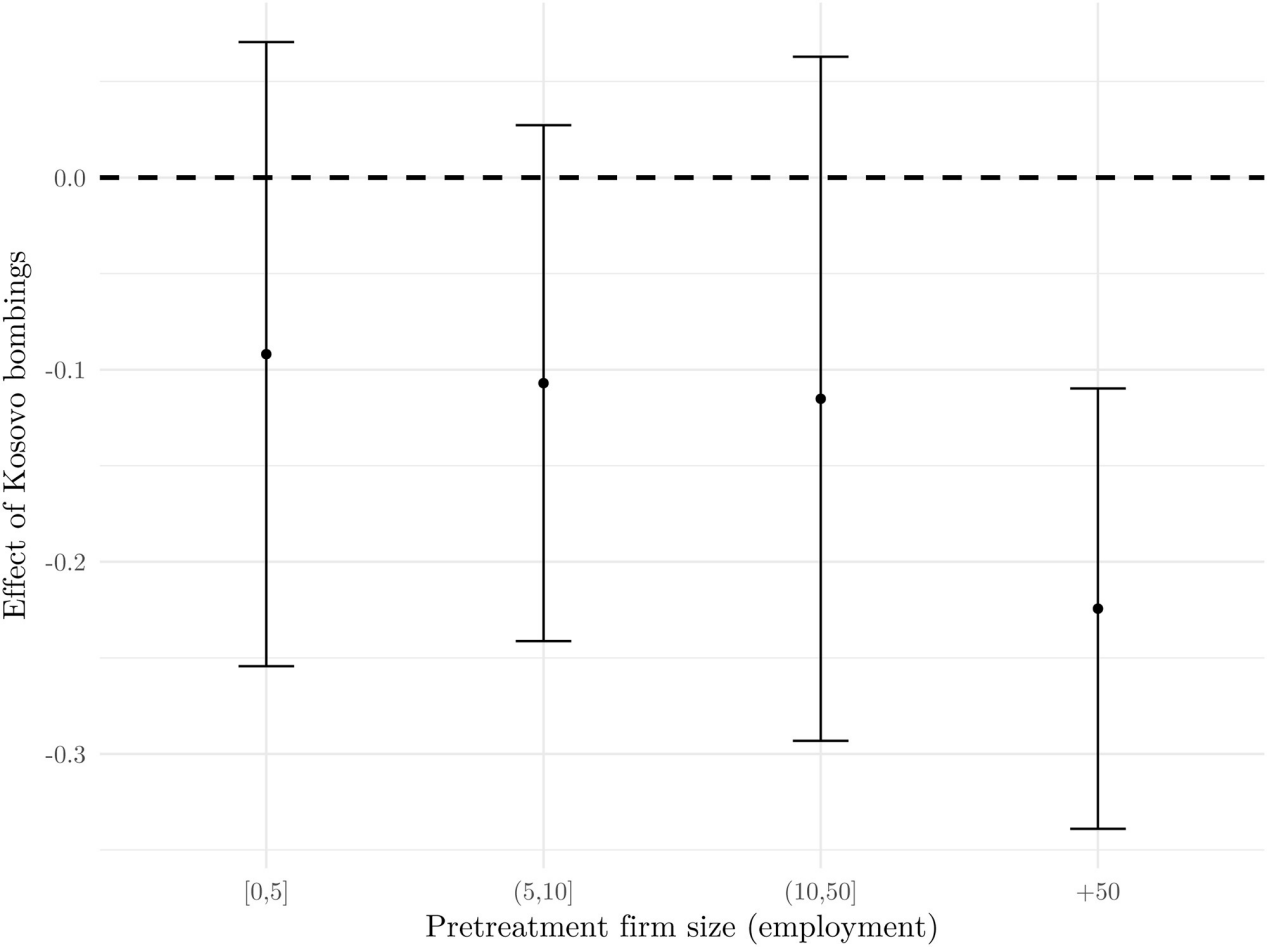
Revenues of manufacturing and tourism industries. Notes: Dots present point estimates (*δ*_*DD*_ from Eq 1), while bars present 95% confidence intervals based on standard errors clustered at the firm level. Estimation is based on the model using the 1998–1999 sample, with municipality and 3-digit NACE fixed effects. Source: The Institute of Economics, Zagreb.

Fig 6 explores heterogeneous effects with respect to the pretreatment firm size in terms of employment. The results indicate that larger firms (50 or more employees) experienced the most drastic drop in revenues. Note that the absence of a significant effect for firms of smaller size might come from larger standard errors due to reduced sample sizes. We also inspect the heterogeneity of the effect with respect to ownership type (private vs. mixed/state-owned) and do not find any significant differences (Figure is omitted due to brevity).

### 5.2 Synthetic control

**Fig 7:**
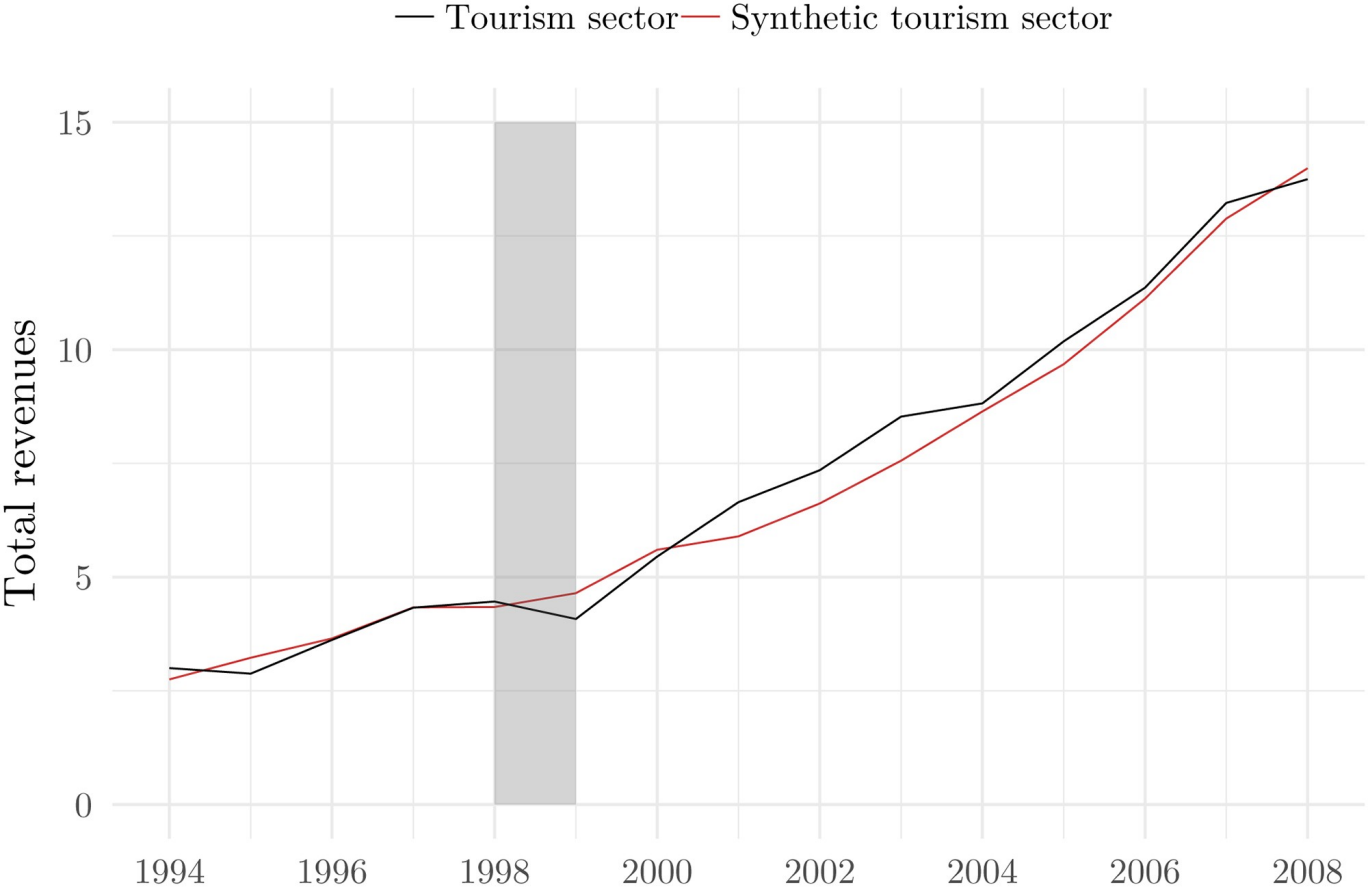
Synthetic control estimation. Notes: Total revenues are in billion Croatian kuna. The model is fitted using data from the 1994–1998 period, after which the synthetic tourism sector is constructed. The shaded gray area represents the time frame of 1999, when the bombings occurred. Source: The Institute of Economics, Zagreb.

In order to explore the long-run consequences of bombing, we combine the original DD estimation strategy with a synthetic control approach developed in [5]. We construct a counterfactual for the tourism sector by collapsing the dataset containing firms from all sectors onto a sectoral level [37]. Using the same set of covariates as in Table 2 (employment, assets and debt) and expanding the time dimension of our sample all the way to 2008, we construct our synthetic tourism sector using data from 18 other economic sectors. Fig 7 reports the results of this exercise which corroborates our baseline results. Apart from that, we also see that the effect is temporary in its nature as the total revenues in the tourism sector recover quickly, already in the following year.

Since the synthetic control was done for a longer time-dimension of the dataset, all the way to 2008 with nine years of post-treatment data, we can discuss the long-run consequences of conflict. Similar to [38] who study the effects of US bombing on long-run development in Vietnam, we also do not find evidence of a prolonged negative effect. A number of other similar studies done mostly for World War II bombings suggest that the negative economic effects vanished rather quickly and that the treated areas returned to their prewar growth trends [39–41].

## 6 Channel of adverse effect: Proximity or perception

So far we have established that NATO bombing has indeed adversely affected the business performance of firms in the tourism sector. As presented in the previous section, this adverse effect is mostly generated in the accommodation service business (hotels) and in firms with 50 or more employees.

In this section we explore whether the channel of proximity is relevant in explaining the intensity of the effect within the country. We take advantage of the specific north-west to south-east orientation of Croatian Adriatic counties in order to analyse the effect of proximity using the following equation:

$$\begin{array}{ll} Y_{istl}= & \alpha +\theta log(dist_{il})+\beta_{1}treat_{is}+\gamma_{1}post_{it}+\beta_{2}(treat_{is}\times log(dist_{il}))+\gamma_{2}(post_{it}\times log(dist_{il}))\\ & +\delta (treat_{is}\times post_{it})+\lambda (treat_{is}\times post_{it}\times log(dist_{il}))+\zeta_{l}+\eta_{s}+e_{istl} \end{array}$$ (2)

where all the variables are the same ones as in Eq 1, with addition of *dist*_*il*_ which represents the geographical distance between firm *i*’s location *l* and the capital of Kosovo—Prishtina. Note that we could have chosen other geographical locations, but the source of heterogeneity would not change, as using different locations to construct proximity would only be a monotonic transformation of the one we use. We construct this variable as the geographical distance between the centeroid of the municipality in which firm *i* reported its headquarters and the geographical location of Prishtina (this implies that location fixed effects *ζ*_*l*_ cannot be at the municipality level as the municipality is used to construct the proximity to NATO bombing—instead we use county fixed effects). Therefore λ represents the intensity of the effect with respect to the proximity to bombing sites. Note that this estimation implies that the intensity is linear in its nature, an assumption we will depart from later on by adopting a more flexible estimation procedure.

**Fig 8:**
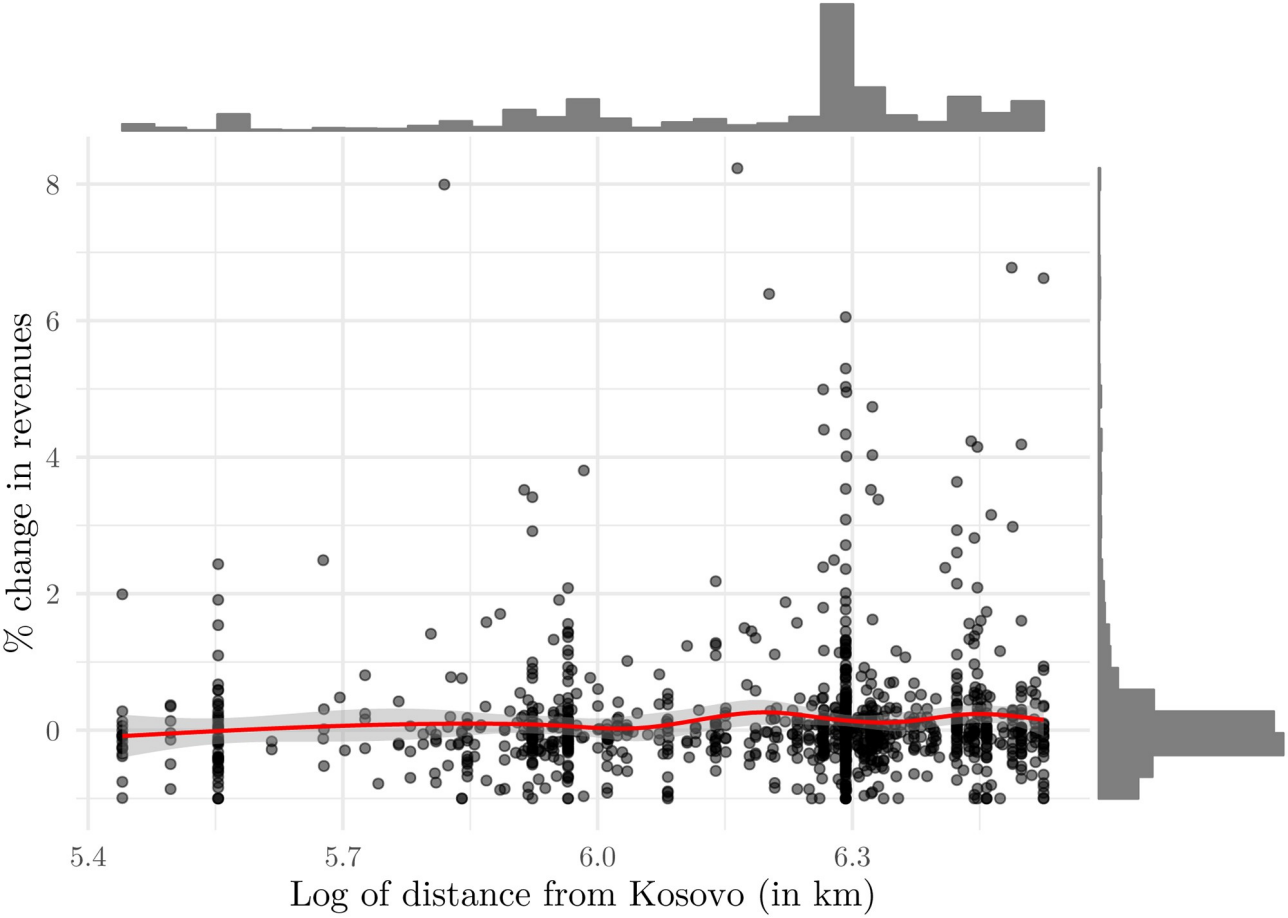
Percentage change in revenues of firms in the tourism sector (1999 vs 1998) and distance from Kosovo. Notes: The red line represents a local linear estimation of the relationship between the percent change in revenues and the log of distance from Kosovo. Gray bars are histograms of percent changes in revenues and log of distance from Kosovo. Source: The Institute of Economics, Zagreb.

Before presenting the results of estimating Eq 2, we present a scatter plot with histograms of log distance from Kosovo in kilometres and percentage change in revenues of firms in the tourism sector from 1998 to 1999 (Fig 8). Distance ranges from 230.5 kilometers, or 5.44 in logs, (Konavle in the Dubrovnik-Neretva county) to 682.5 kilometres (Umag in the Istria county), or 6.53 in logs, both of which are municipalities largely engaged in tourism activities. The spikes in the mass observed in Fig 8 come from the concentration of economic activity in particular locations. For example, firms are concentrated in and around the largest three cities in Croatia: Split (389 km distance, 5.96 in logs), Zagreb (540 km distance, 6.29 in logs) and Rijeka (615 km distance, 6.42 in logs) which is reflected by spikes in the histogram of distance. Fig 8 already indicates what Tables 5 and 6 will show in detail—distance within Croatia is not the channel of an adverse effect.

**Table 5:** Intensity of the effect with respect to distance.

	*Dependent variable: log revenues*					
	*Whole Croatia*			*Adriatic Croatia*		
	(1)	(2)	(3)	(4)	(5)	(6)
Treated sector x Treated time x log(Distance)	0.299	0.281	0.334	−0.048	−0.009	0.017
	(0.320)	(0.319)	(0.317)	(0.385)	(0.384)	(0.383)
Treated sector x Treated time	−2.024	−1.899	−2.244	0.003	−0.238	−0.418
	(1.984)	(1.976)	(1.962)	(2.378)	(2.373)	(2.367)
Treated sector x log(Distance)	−1.185***	−0.771**	−1.000***	−1.078**	−0.797*	−0.851**
	(0.396)	(0.390)	(0.363)	(0.465)	(0.462)	(0.433)
Treated time x log(Distance)	0.036	0.055	0.027	0.292	0.300	0.308
	(0.198)	(0.197)	(0.195)	(0.259)	(0.256)	(0.256)
Treated sector	6.585***	3.921	5.444**	6.092**	4.242	4.677*
	(2.460)	(2.435)	(2.267)	(2.873)	(2.872)	(2.694)
Treated time	−0.265	−0.380	−0.175	−1.750	−1.782	−1.801
	(1.237)	(1.232)	(1.219)	(1.612)	(1.592)	(1.592)
log(Distance)	0.297	0.432*	0.540**	0.325	0.463	0.421
	(0.239)	(0.238)	(0.221)	(0.299)	(0.296)	(0.278)
3-digit NACE code effects	No	Yes	Yes	No	Yes	Yes
Employment, assets and debt	No	No	Yes	No	No	Yes
Observations	14,177	14,177	14,131	4,714	4,714	4,700

Notes: Treated sector x Treated period x log(Distance) is λ from Eq 2. Estimation includes the 1998–1999 period for all models. Estimates using the whole Croatia sample include all 21 Croatian counties, while Adriatic Croatia includes seven Adriatic counties. Standard errors clustered at the firm level are in brackets.

*p<0.1;

**p<0.05;

***p<0.01

**Table 6:** Intensity of the effect with respect to distance: Tourism sector only.

	*Dependent variable: log revenues*					
	*Whole Croatia*			*Adriatic Croatia*		
	(1)	(2)	(3)	(4)	(5)	(6)
Treated time x log(Distance)	0.336	0.334	0.365	0.243	0.287	0.321
	(0.251)	(0.252)	(0.251)	(0.285)	(0.286)	(0.286)
Treated time	−2.288	−2.265	−2.441	−1.748	−1.990	−2.204
	(1.551)	(1.552)	(1.547)	(1.751)	(1.758)	(1.758)
log(Distance)	−0.888***	−0.386	−0.489*	−0.752**	−0.394	−0.463
	(0.316)	(0.312)	(0.294)	(0.357)	(0.356)	(0.336)
3-digit NACE code effects	No	Yes	Yes	No	Yes	Yes
Employment, assets and debt	No	No	Yes	No	No	Yes
Observations	2,716	2,716	2,712	1,401	1,401	1,397

Notes: Estimation includes the 1998–1999 period for all models, only tourism sector is included. Estimates using the whole Croatia sample include all 21 Croatian counties, while Adriatic Croatia includes seven Adriatic counties. Standard errors clustered at the firm level are in brackets.

*p<0.1;

**p<0.05;

***p<0.01

Results for Eq 2 are presented in Table 5. Columns (1) to (3) present the results for whole Croatia, while columns (4) to (6)—where we exploit the northwest-southeast orientation of the Croatian coast—contain only models which use the seven Adriatic counties. Models also differ in the inclusion of covariates. The results indicate that the intensity of the effect of NATO bombing on revenues of firms in the tourism sector does not significantly vary with distance from the bombing sites. The estimated effect cannot be statistically distinguished from zero in either of the specifications. Estimation of the intensity of the effect with respect to the distance from Kosovo based only on the tourism sector—presented in Table 6—corroborates these findings.

**Fig 9:**
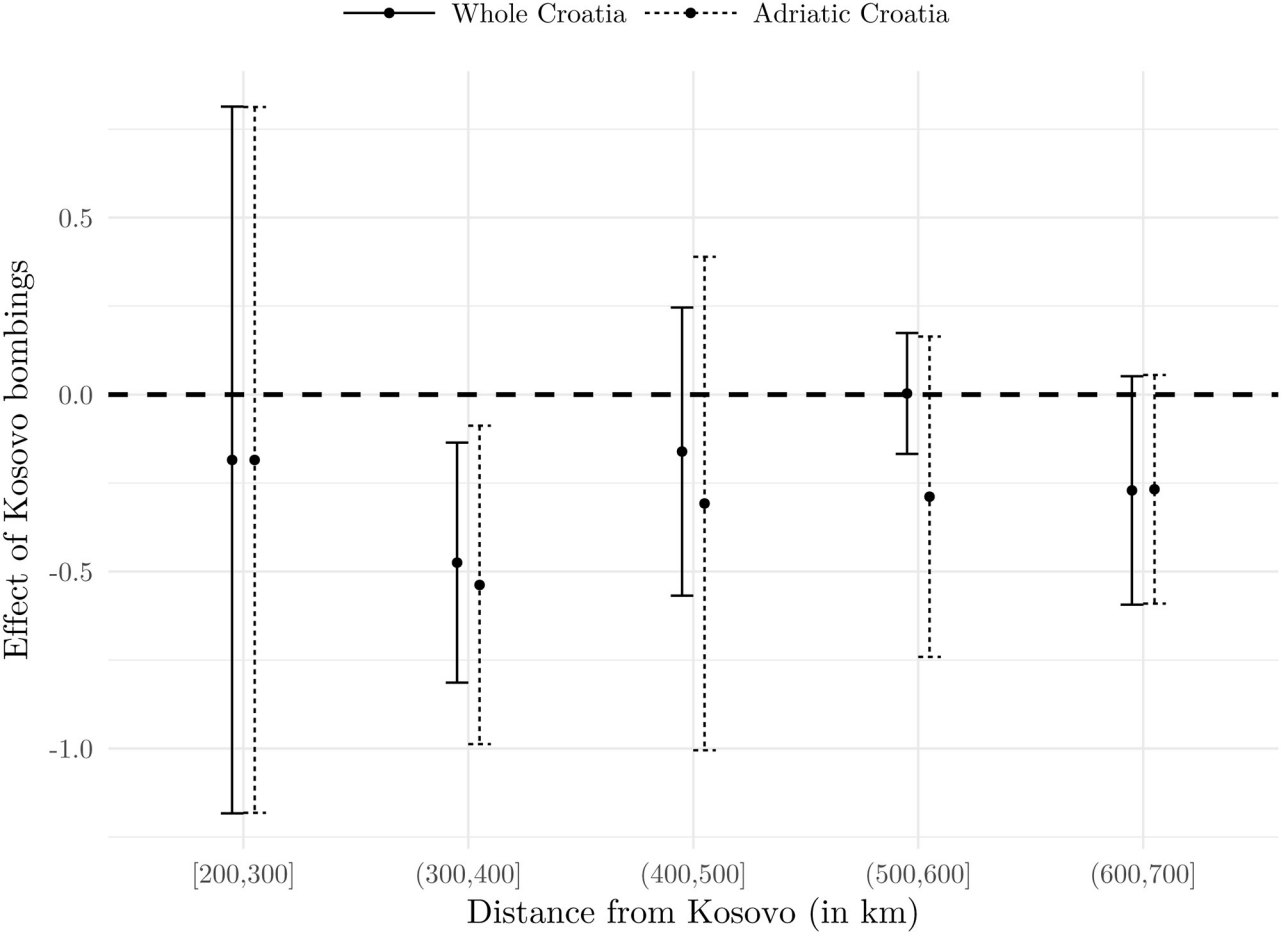
Distance from the bombings and the Size of the Effect. Notes: Dots present point estimates (*δ*_*DD*_ from Eq 1), while bars present 95% confidence intervals based on standard errors clustered at the firm level. Estimation is based on the model using the 1998–1999 sample, with county and 3-digit NACE fixed effects. Source: The Institute of Economics, Zagreb.

**Fig 10:**
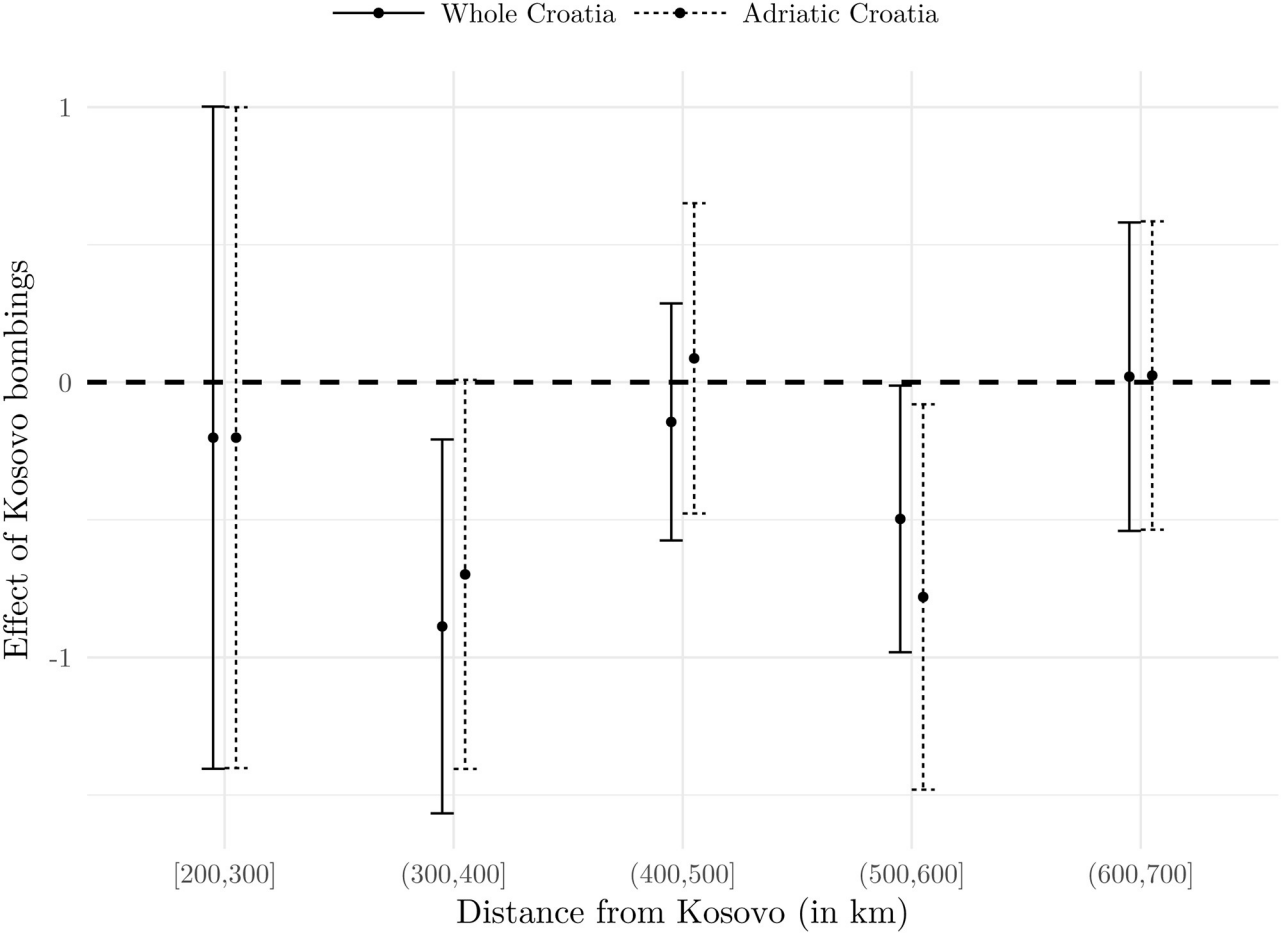
Distance from the bombings and the Size of the Effect: Accommodation. Notes: Dots present point estimates (*δ*_*DD*_ from Eq 1), while bars present 95% confidence intervals based on standard errors clustered at the firm level. Estimation is based on the model using the 1998–1999 sample, with county fixed effects. Source: The Institute of Economics, Zagreb.

In order to further explore the nexus between the proximity and the adverse effect, we split our sample according to the distance from Prishtina and run our preferred specification. This enables us to explore this channel in a more flexible manner. Results for whole Croatia and for the Adriatic Croatia are presented in Figs 9 and 10, and they indicate that no significant heterogeneity, with respect to distance, is recorded. (we actually run the analysis for NATO’s first targets: Prishtina in Kosovo, Belgrade—the capital of Serbia and Yugoslavia—Kragujevac in Serbia, and Podgorica in Montenegro as detected in [42]. The targets were airports, military barracks, an aircraft factory, radar stations, an air base and an arms factory. All four estimation results are similar to the Prishtina case and are available upon request from the authors). Although point estimates do differ, indicating that the strongest adverse effects were experienced by firms with headquarters 300 to 400 kilometres from Prishtina, the fact that the 95 percent confidence intervals overlap in each case suggests that we cannot reject the hypothesis that proximity is not relevant in explaining the intensity of the effect. While this might come from relatively high standard errors, we argue this evidence is compelling enough to conclude that within-country proximity to conflict is not a significant channel of the adverse effect.

**Fig 11:**
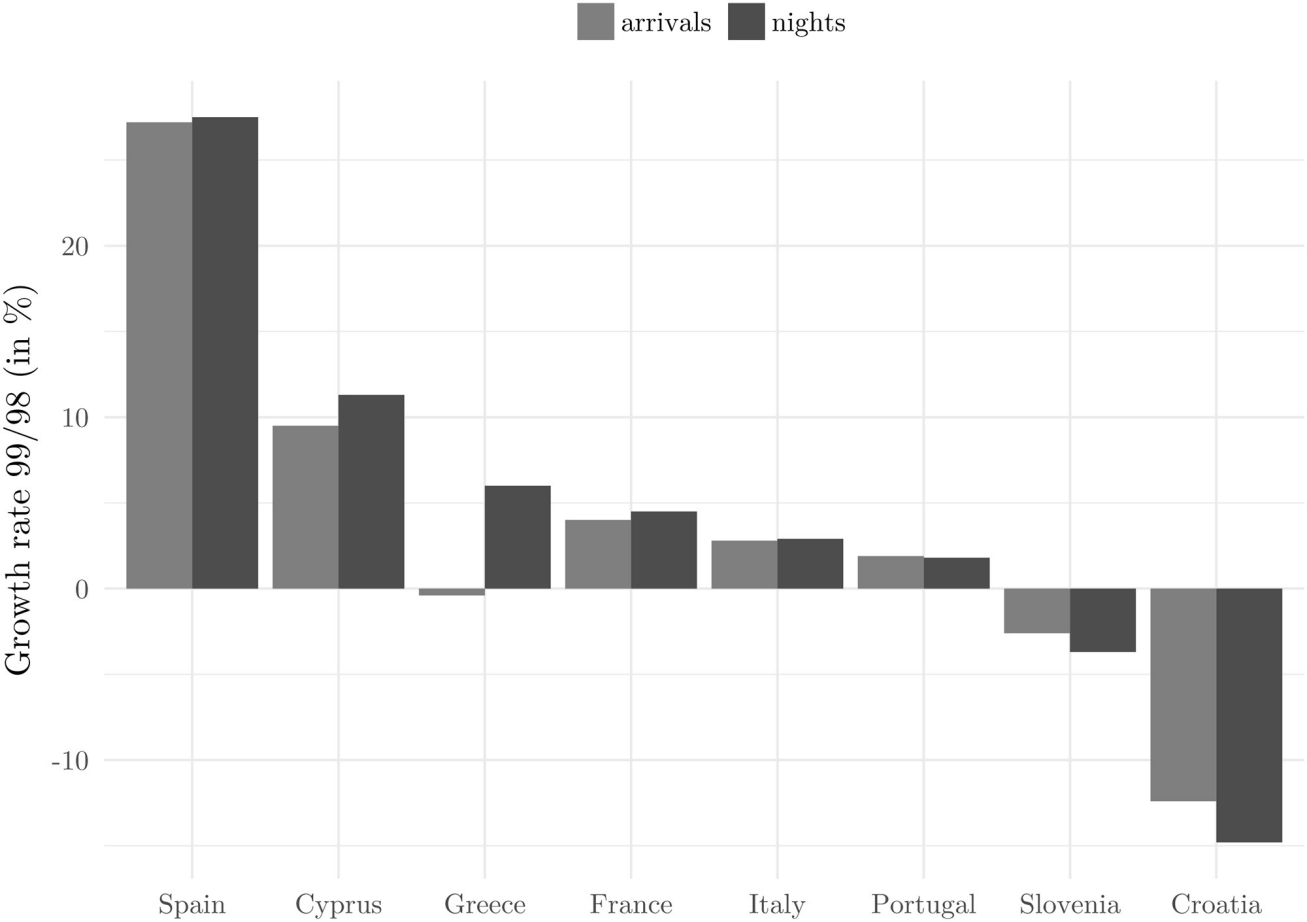
Tourist arrivals and nights spent: Competition. Source: Eurostat.

Other evidence of the claim that proximity is not a significant channel of the adverse effect comes from outside Croatian borders. It seems that only one other competitor country (out of a pool of countries for which we have data) suffered losses during 1999. That country was Slovenia and in that year it lost around 45,000 tourists (Fig 11). Although the size of the impact in Slovenia was much smaller than in Croatia, we have to take into account that Slovenia is not as big a tourist destination as Croatia. All other countries from our sample recorded significant increases in the number of tourist arrivals and nights spent. These developments suggest that Slovenia as well as Croatia was perceived as an insecure destination, possibly as both countries were part of Yugoslavia (a country then being bombed by NATO) just eight years earlier.

Fig 11 also corroborates the absence of the 1998 Russian financial crisis effect on tourism in general. According to the figures for Spain, Cyprus, Greece, France, Italy, and Portugal, the Russian financial crisis did not have an adverse influence in 1998 as the recovery was swift.

## 7 Conclusion

In this paper we have tried to illuminate the impact of conflicts on consumer behaviour on the tourism market. Although we do a single-country study and explore the Croatian tourism sector, we find that our study is relevant in an international context as well. Due to frequent crisis events in popular tourist destinations (e.g. attacks in Tunisia, Turkey, France, Spain, etc.), we believe our research can provide useful insights, despite the fact that we explore the impacts of a longer-term conflict that culminated in NATO military intervention.

The results we obtain are expected—there is a large negative effect of conflict on tourism—but we contribute to the literature by exploring whether the adverse effects within the country are channelled through proximity to conflict areas. By analysing heterogeneous effects with respect to the distance of the firm from Kosovo, we argue that within-country proximity to conflict is not a significant channel through which the negative effect propagates. This essentially suggests that conflict or possibly even war, or an act of terrorism, has far-reaching effects that go beyond simple geographical proximity to the conflict. There are obviously other channels that drive tourists away form certain destinations, one of which may possibly be perception. We speculate that foreign tourists could have perceived Croatia as part of an unstable region as only a few years had passed since 1991 and the violent breakup of Yugoslavia. This view, driven by conflict more than 600 kilometres away, potentially led them away from Croatia as a tourism destination. Unfortunately, there is not much information in support of our hypothesis, besides simple press articles. For example, an article from The New York Times from April 1999 argues that Croatia “faces a bleak summer of empty hotels and beaches” and that “the assaults appear to have shattered prospects for the country’s tourist industry this year” [43, 44]. Reports a special analysis on the economic consequences of the Kosovo conflict for neighboring countries. It emphasizes that the crisis in Kosovo adversely affected investors” sentiment and that normal earning of tourism revenues in Croatia is jeopardized. Finally [30], reports that one of the main channels of the impact of the Kosovo crisis on Croatia is the damage to consumer and investor confidence that will result in a significant loss in tourist receipts. A report published four months later [31], in the aftermath of the crisis, argues that although the crisis deterred tourists from the region, it seems to be only short-lived as reports of a rebound in booking have already been reported (mostly in Bulgaria but also to a lesser extent in Croatia). Unfortunately, no specific surveys from that time target the region. We believe this is due to the fact that the NATO strike was sudden and short-lived so there was probably no time to implement the specific questions into the surveys.

Apart from the novel channel of adverse effect of conflict on tourism, we also find this type of identification-based empirical work useful in tourism research. Using the difference-in-differences and the synthetic control identification strategy enabled us to measure the causal effect on a tourism outcome, not just simple correlations, as is normally the case in tourism research. The approach taken here allows us to answer the “what if” type of questions, e.g.: “How many tourist arrivals would there have been if NATO had not bombed Kosovo?”. We believe that this research is just the beginning of extended use of identification-based empirical work in tourism research as the benefits are large—from valuable policy applications to deeper understanding of tourism.

## Supporting information

S1 Appendix (PDF)
