# Supplementary material for: Does proximity to conflict affect tourism: Evidence from NATO bombing
Source: PLoS One. 2021 Oct 20;16(10):e0258195. doi: 10.1371/journal.pone.0258195 (PMC8528334; doi:10.1371/journal.pone.0258195)
Supplement: S1 Appendix — (PDF) [file pone.0258195.s001.pdf]

8 Appendix

Table 7: Kosovo Bombings and Tourism: Different Levels of Clustered Standard Errors

	Dependent variable: log revenues					
	1993–1999 sample			*1998–1999 sample*		
	(1)	(2)	(3)	(4)	(5)	(6)
Treated sector x Treated period	−0.234	−0.191	−0.203	−0.173	−0.166	−0.180
Clustered standard errors:						
At the firm level	(0.076)***	(0.076)**	(0.074)***	(0.070)**	(0.071)**	(0.071)**
At the NACE level	(0.111)**	(0.072)***	(0.062)***	(0.083)**	(0.078)**	(0.081)**
At the county level	(0.091)**	(0.077)**	(0.077)**	(0.072)**	(0.065)**	(0.066)**
At the municipal level	(0.074)***	(0.070)***	(0.071)***	(0.062)***	(0.060)***	(0.059)***
At the municipal X NACE level	(0.076)***	(0.071)***	(0.068)***	(0.067)**	(0.067)**	(0.066)***
Treated sector and period	Yes	Yes	Yes	Yes	Yes	Yes
Other covariates	No	No	Yes	No	No	Yes
Municipality fixed effects	No	Yes	Yes	No	Yes	Yes
3-digit NACE code effects	No	Yes	Yes	No	Yes	Yes
Observations	38,463	38,463	35,482	14,177	14,177	14,131

Note: Other covariates include employment count, long-term assets and long-term debt.

*p<0.1; **p<0.05; ***p<0.01
